# Complete genome sequence of *Sulfurospirillum deleyianum* type strain (5175^T^)

**DOI:** 10.4056/sigs.671209

**Published:** 2010-03-24

**Authors:** Johannes Sikorski, Alla Lapidus, Alex Copeland, Tijana Glavina Del Rio, Matt Nolan, Susan Lucas, Feng Chen, Hope Tice, Jan-Fang Cheng, Elizabeth Saunders, David Bruce, Lynne Goodwin, Sam Pitluck, Galina Ovchinnikova, Amrita Pati, Natalia Ivanova, Konstantinos Mavromatis, Amy Chen, Krishna Palaniappan, Patrick Chain, Miriam Land, Loren Hauser, Yun-Juan Chang, Cynthia D. Jeffries, Thomas Brettin, John C. Detter, Cliff Han, Manfred Rohde, Elke Lang, Stefan Spring, Markus Göker, Jim Bristow, Jonathan A. Eisen, Victor Markowitz, Philip Hugenholtz, Nikos C. Kyrpides, Hans-Peter Klenk

**Affiliations:** 1 DSMZ - German Collection of Microorganisms and Cell Cultures GmbH, Braunschweig, Germany; 2 DOE Joint Genome Institute, Walnut Creek, California, USA; 3 Los Alamos National Laboratory, Bioscience Division, Los Alamos, New Mexico, USA; 4 Biological Data Management and Technology Center, Lawrence Berkeley National Laboratory, Berkeley, California, USA; 5 Lawrence Livermore National Laboratory, Livermore, California, USA; 6 Oak Ridge National Laboratory, Oak Ridge, Tennessee, USA; 7 HZI - Helmholtz Centre for Infection Research, Braunschweig, Germany; 8 University of California Davis Genome Center, Davis, California, USA

**Keywords:** anaerobic, microaerobic, sulfur reduction, dissimilatory nitrate reduction, Gram-negative, motile, *Campylobacteraceae*, GEBA

## Abstract

*Sulfurospirillum deleyianum* Schumacher *et al.* 1993 is the type species of the genus *Sulfurospirillum*. *S. deleyianum* is a model organism for studying sulfur reduction and dissimilatory nitrate reduction as an energy source for growth. Also, it is a prominent model organism for studying the structural and functional characteristics of cytochrome c nitrite reductase. Here, we describe the features of this organism, together with the complete genome sequence and annotation. This is the first completed genome sequence of the genus *Sulfurospirillum*. The 2,306,351 bp long genome with its 2,291 protein-coding and 52 RNA genes is part of the *** G****enomic* *** E****ncyclopedia of* *** B****acteria and* *** A****rchaea * project.

## Introduction

Strain 5175^T^ (= DSM 6946 = ATCC 51133 = LMG 8192) is the type strain of the species *Sulfurospirillum deleyianum*, which is the type species of the genus *Sulfurospirillum*. The genus *Sulfurospirillum* was originally proposed by Schumacher *et al.* in 1992 [1]. The generic name *Sulfurospirillum* derives from the chemical element ‘sulfur’ and ‘spira’ from Latin meaning coil, a coiled bacterium that reduces sulfur [2]. The species is named after J. De Ley, a Belgian microbiologist who significantly contributed to bacterial systematics based on genetic relationships [3]. Altogether, the genus *Sulfurospirillum* contains seven species [2]. Strain 5175^T^ was isolated from anoxic mud of a forest pond near Heinigen, Braunschweig area, Germany [3]. It is unclear if further isolates of the species exist. Here, we present a summary classification and a set of features for *S. deleyianum* 5175^T^, together with the description of the complete genomic sequencing and annotation.

## Classification and features

There were several uncultured clone sequences known in INSDC databases with at least 98% sequence identity to the 16S rRNA gene sequence (Y13671) of strain *S. deleyianum* 5175^T^. These were obtained from lake material in Dongping, China (FJ612333), deep subsurface groundwater in Japan (AB237694), and from the mangrove ecosystem of the Danshui River Estuary of Northern Taiwan (DQ234237) [4]. No significant matches were reported with metagenomic samples at the NCBI BLAST server (November 2009).

**Figure 1:**
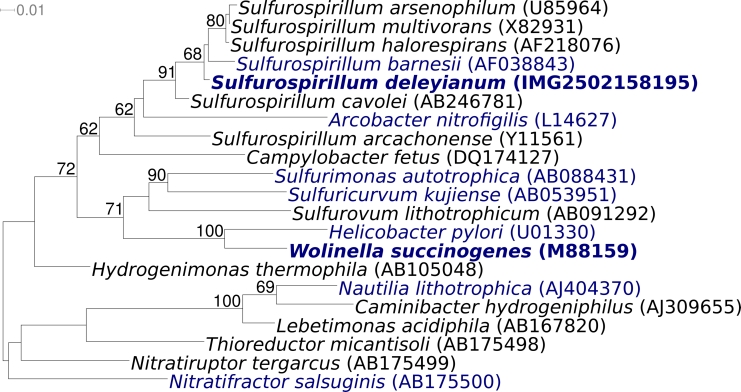
Phylogenetic tree highlighting the position of *S. deleyianum* 5175^T^ relative to the other type strains within the genus and the type strains of the other genera within the class *Epsilonproteobacteria.* The tree was inferred from 1,326 aligned characters [5,6] of the 16S rRNA gene sequence under the maximum likelihood criterion [7] and rooted with the *Nautiliales*. The branches are scaled in terms of the expected number of substitutions per site. Numbers above branches are support values from 450 bootstrap replicates if larger than 60%. Lineages with type strain genome sequencing projects registered in GOLD [8] are shown in blue, published genomes in bold.

Figure 1 shows the phylogenetic neighborhood of *S. deleyianum* 5175^T^ in a 16S rRNA based tree. The sequences of the three 16S rRNA gene copies in the genome of *S. deleyianum* 5175^T^ differ from each other by no more than one nucleotide, and differ by no more than one nucleotide from the previously published 16S rRNA sequence (Y13671).

**Figure 2:**
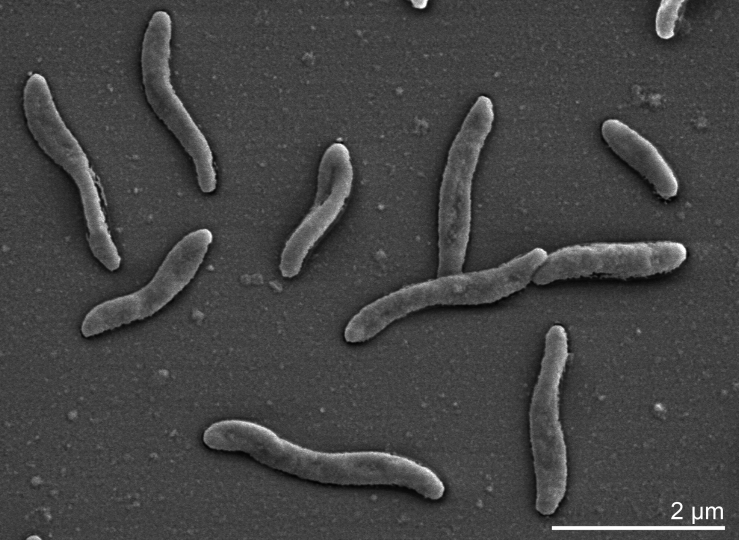
Scanning electron micrograph of *S. deleyianum* 5175^T^

The cells of strain 5175^T^ are curved spiral rods of approximately 0.3-0.5 µm width and 1.0-3.0 µm length [1], with polar flagellation (Table 1 and Figure 2). Colonies are yellow-colored as a result of a flexirubin-type pigment [17]. The cells contain cytochrome b and c [4]. Strain 5175^T^ is unable to rapidly decompose H_2_O_2_ (i.e. is catalase negative), does not need special growth factors (vitamins or amino acids), and is positive for oxidase [1]. Strain 5175^T^ grows anoxically with hydrogen, formate, fumarate, and pyruvate, but not lactate, as electron donor; acetate and hydrogen carbonate as carbon source and one of the following electron acceptors: nitrate, nitrite (which is reduced to ammonia), sulfite, thiosulfate, elemental sulfur (reduced to sulfide), dimethyl sulfoxide (reduced to dimethyl sulfide), fumarate, malate and aspartate (reduced to succinate) [1,18]. Sulfate is not reduced. Fumarate and malate can be fermented [1]. Strain 5175^T^ is able to grow microaerobically at 1-4% oxygen, but not at 21% oxygen [1]. The substrates utilized for microaerobic growth are succinate, fumarate, malate, aspartate, pyruvate, oxoglutarate, and oxaloacetate [1]. There is no oxidation of glycerol or acetate [1]. An assimilatory sulfate reduction is lacking, and a source of reduced sulfur, *e.g.* sulfide [19] or L-cysteine, is required for growth [1]. Further characteristics of the sulfur respiration of strain 5175^T^ have been studied in detail [4,20].

**Table 1:** Classification and general features of *S. deleyianum* 5175^T^ according to the MIGS recommendations [9]

**MIGS ID**	**Property**	**Term**	**Evidence code**
	Current classification	Domain *Bacteria*	TAS [10]
		Phylum *Proteobacteria*	TAS [11]
		Class *Epsilonproteobacteria*	TAS [12]
		Order *Campylobacterales*	TAS [12]
		Family *Campylobacteraceae*	TAS [13]
		Genus *Sulfurospirillum*	TAS [1]
		Species *Sulfurospirillum deleyianum*	TAS [1]
		Type strain 5175	TAS [1]
	Gram stain	negative	TAS [1]
	Cell shape	curved spiral rods	TAS [1]
	Motility	motile by polar flagellum	TAS [1]
	Sporulation	non-sporulating	TAS [1]
	Temperature range	20°C-36°C, no growth at 42°C	TAS [14]
	Optimum temperature	30°C	NAS
	Salinity	< 0.2%	TAS [14]
MIGS-22	Oxygen requirement	anaerobic, microaerobic (1-4% oxygen)	TAS [1]
	Carbon source	dicarboxylic acids, aspartate, pyruvate, acetate, hydrogen carbonate	TAS [1]
	Energy source	dicarboxylic acids, aspartate, pyruvate, formate, H_2_, H_2_S	TAS [1,3]
MIGS-6	Habitat	anoxic mud	TAS [1]
MIGS-15	Biotic relationship	free living	TAS [1]
MIGS-14	Pathogenicity	none	NAS
	Biosafety level	1	TAS [15]
	Isolation	anoxic mud from a German lake	TAS [1]
MIGS-4	Geographic location	Heinigen near Wolfenbüttel	TAS [3]
MIGS-5	Sample collection time	1976	NAS
MIGS-4.1 MIGS-4.2	Latitude Longitude	52.17 10.55	NAS
MIGS-4.3	Depth	not reported	
MIGS-4.4	Altitude	not reported	

Evidence codes - IDA: Inferred from Direct Assay (first time in publication); TAS: Traceable Author Statement (i.e., a direct report exists in the literature); NAS: Non-traceable Author Statement (i.e., not directly observed for the living, isolated sample, but based on a generally accepted property for the species, or anecdotal evidence). These evidence codes are from of the Gene Ontology project [16]. If the evidence code is IDA, then the property should have been directly observed by one of the authors or an expert mentioned in the acknowledgements.

Observations of ferric iron-reducing bacteria indicated that ferrihydrite was reduced to ferrous iron minerals via sulfur cycling with sulfide as the reductant. Ferric iron reduction via sulfur cycling was investigated in more detail with strain 5175^T^, which can utilize sulfur or thiosulfate as an electron acceptor [21]. In the presence of cysteine (0.5 or 2 mM) as the sole sulfur source, no (microbial) reduction of ferrihydrite or ferric citrate was observed, indicating that *S. deleyianum* is unable to use ferric iron as an immediate electron acceptor [21]. Interestingly, with thiosulfate at low concentration (0.05 mM), growth with ferrihydrite (6 mM) was possible, and sulfur was cycled up to 60 times [21].

An interesting syntrophism between strain 5175^T^ and *Chlorobium limicola* 9330 has been reported [22]. The substrate formate is not metabolized by *Chlorobium*, and the limiting amount of sulfur is alternately reduced by strain 5175^T^ and oxidized by *Chlorobium* [22].

With respect to utilization of nitrate as terminal electron acceptor (dissimilatory nitrate reduction) [3] the dissimilatory hexaheme *c* nitrite has been studied in more detail. These include both structural and functional aspects [23-26].

Also, strain 5175^T^ is able to use alternative electron acceptors. Strain 5175^T^ is able to reduce the quinone moiety of anthraquinone-2,6-disulfonate (AQDS) and also to oxidize reduced anthrahydroquinone-2,6,-disulfonate (AH2QDS) as well [27]. Additionally, oxidized metals may be used as terminal electron acceptors, such as arsenate [As(V)] and manganese [Mn(IV)], but not selenate [Se(VI)] or ferric iron [Fe(III)] [27].

### Chemotaxonomy

The predominant menaquinone is MK-6 (88%), with small amounts of thermoplasmaquinone with six isoprene units (TPQ-6; 10%) and MK-5 (2%) [28]. The polar-lipid fatty acid composition is 16:1ω7c (52.0%), 16:0 (29.2%), 18:1ω7c (17.2%), 15:0 (1.1%), and iso16:1 (0.6%) [29].

## Genome sequencing and annotation

### Genome project history

This organism was selected for sequencing on the basis of its phylogenetic position, and is part of the *** G****enomic* *** E****ncyclopedia of* *** B****acteria and* *** A****rchaea * project [31]. The genome project is deposited in the Genomes OnLine Database [10] and the complete genome sequence is deposited in GenBank. Sequencing, finishing and annotation were performed by the DOE Joint Genome Institute (JGI). A summary of the project information is shown in Table 2.

**Table 2:** Genome sequencing project information

**MIGS ID**	**Property**	**Term**
MIGS-31	Finishing quality	Finished
MIGS-28	Libraries used	One Sanger libraries 8 kb pMCL200 and One 454 pyrosequence standard library
MIGS-29	Sequencing platforms	ABI3730, 454 GS FLX
MIGS-31.2	Sequencing coverage	9.12× Sanger, 25.3× pyrosequence
MIGS-30	Assemblers	Newbler, phrap
MIGS-32	Gene calling method	Prodigal, GenePRIMP
	INSDC ID	CP001816
	Genbank Date of Release	November 18, 2009
	GOLD ID	Gc01143
	NCBI project ID	29529
	Database: IMG-GEBA	2502082112
MIGS-13	Source material identifier	DSM 6946
	Project relevance	Tree of Life, GEBA

### Growth conditions and DNA isolation

*S. deleyianum* 5175^T^, DSM 6946, was grown anaerobically in DSM medium 541 [30] at 28°C. DNA was isolated from 0.5-1 g of cell paste using Qiagen Genomic 500 DNA Kit (Qiagen, Hilden, Germany) following the manufacturer’s protocol with modification st/L for cell lysis as described in Wu *et al.* [31].

### Genome sequencing and assembly

The genome was sequenced using a combination of Sanger and 454 sequencing platforms. All general aspects of library construction and sequencing can be found at http://www.jgi.doe.gov/. 454 Pyrosequencing reads were assembled using the Newbler assembler version 1.1.02.15 (Roche). Large Newbler contigs were broken into 2,525 overlapping fragments of 1,000 bp and entered into assembly as pseudo-reads. The sequences were assigned quality scores based on Newbler consensus q-scores with modifications to account for overlap redundancy and to adjust inflated q-scores. A hybrid 454/Sanger assembly was made using the phrap assembler. Possible mis-assemblies were corrected with Dupfinisher or transposon bombing of bridging clones [32]. Gaps between contigs were closed by editing in Consed, custom primer walk or PCR amplification. A total of 471 Sanger finishing reads were produced to close gaps, to resolve repetitive regions, and to raise the quality of the finished sequence. The error rate of the completed genome sequence is less than 1 in 100,000. Together all sequence types provided 34.42× coverage of the genome. The final assembly contains 23,491 Sanger and 296,611 pyrosequence reads.

### Genome annotation

Genes were identified using Prodigal [33] as part of the Oak Ridge National Laboratory genome annotation pipeline, followed by a round of manual curation using the JGI GenePRIMP pipeline [34]. The predicted CDSs were translated and used to search the National Center for Biotechnology Information (NCBI) nonredundant database, UniProt, TIGRFam, Pfam, PRIAM, KEGG, COG, and InterPro databases. Additional gene prediction analysis and manual functional annotation was performed within the Integrated Microbial Genomes Expert Review (IMG-ER) platform [35].

## Genome properties

**Figure 3:**
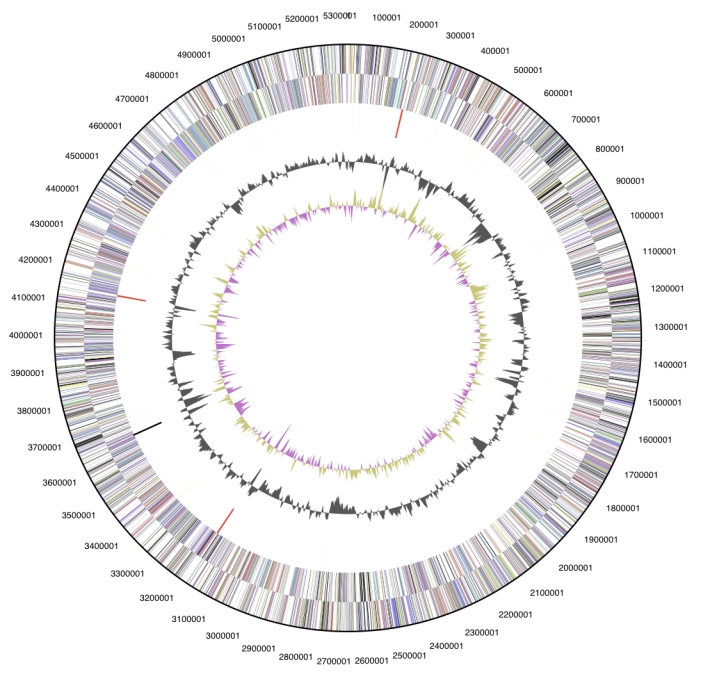
Graphical circular map of the genome. From outside to the center: Genes on forward strand (color by COG categories), Genes on reverse strand (color by COG categories), RNA genes (tRNAs green, rRNAs red, other RNAs black), GC content, GC skew.

The genome consists of a 2,306,351 bp long chromosome with a 39.0% GC content (Table 3 and Figure 3). Of the 2,343 genes predicted, 2,291 were protein coding genes, and 52 RNAs. A total of 26 pseudogenes were identified. The majority of the protein-coding genes (72.9%) were assigned with a putative function while those remaining were annotated as hypothetical proteins. The distribution of genes into COGs functional categories is presented in Table 4.

**Table 3:** Genome Statistics

**Attribute**	**Value**	**% of Total**
Genome size (bp)	2,306,351	100.00%
DNA coding region (bp)	2,171,873	94.17%
DNA G+C content (bp)	898,781	38.97%
Number of replicons	1	
Extrachromosomal elements	0	
Total genes	2,343	100.00%
RNA genes	52	2.22%
rRNA operons	3	
Protein-coding genes	2,291	97.78%
Pseudo genes	26	1.11%
Genes with function prediction	1,708	72,90%
Genes in paralog clusters	254	10.84%
Genes assigned to COGs	1,724	73.58%
Genes assigned Pfam domains	1,750	74.69%
Genes with signal peptides	439	18.74%
Genes with transmembrane helices	566	24.16%
CRISPR repeats	2	

**Table 4:** Number of genes associated with the general COG functional categories

**Code**	**value**	**%age**	**Description**
J	141	6.2	Translation, ribosomal structure and biogenesis
A	0	0.0	RNA processing and modification
K	88	3.8	Transcription
L	113	4.9	Replication, recombination and repair
B	0	0.0	Chromatin structure and dynamics
D	25	1.1	Cell cycle control, mitosis and meiosis
Y	0	0.0	Nuclear structure
V	27	1.1	Defense mechanisms
T	181	7.9	Signal transduction mechanisms
M	128	5.6	Cell wall/membrane biogenesis
N	83	3.6	Cell motility
Z	0	0.0	Cytoskeleton
W	0	0.0	Extracellular structures
U	61	2.7	Intracellular trafficking and secretion
O	85	3.7	Posttranslational modification, protein turnover, chaperones
C	150	6.5	Energy production and conversion
G	53	2.3	Carbohydrate transport and metabolism
E	154	6.7	Amino acid transport and metabolism
F	51	2.2	Nucleotide transport and metabolism
H	101	4.4	Coenzyme transport and metabolism
I	43	1.9	Lipid transport and metabolism
P	112	4.9	Inorganic ion transport and metabolism
Q	21	0.9	Secondary metabolites biosynthesis, transport and catabolism
R	194	8.5	General function prediction only
S	119	5.2	Function unknown
-	619	27.0	Not in COGs
